# A novel algorithm for complete ranking of DMUs dealing with negative data using Data Envelopment Analysis and Principal Component Analysis: Pharmaceutical companies and another practical example

**DOI:** 10.1371/journal.pone.0290610

**Published:** 2023-09-01

**Authors:** Hoda Dalili Yazdi, Farzad Movahedi Sobhani, Farhad Hosseinzadeh Lotfi, Hamed Kazemipoor

**Affiliations:** 1 Department of Industrial Engineering, Science and Research Branch, Islamic Azad University, Tehran, Iran; 2 Department of Mathematics, Science and Research Branch, Islamic Azad University, Tehran, Iran; 3 Department of Industrial Engineering, Central Tehran Branch, Islamic Azad University, Tehran, Iran; Shandong University of Science and Technology, CHINA

## Abstract

When there is an extensive number of inputs and outputs compared to the number of DMUs, one of the drawbacks of Data Envelopment Analysis appears, which incorrectly classifies inefficient DMUs, as efficient ones. Accordingly, the DEA ranking power becomes further moderated. To improve the ranking power, this paper renders the details of an algorithm that presents a model combining the Principal Component Analysis and the Slacks-Based Measure (PCA-SBM) which reduces the number of the incorrectly determined efficient DMUs. Also to complete ranking of DMUs, the algorithm presents a Super-Efficiency model integrated with PCA (PCA-Super SBM) which can rank the efficient DMUs (extreme and non-extreme). Whereas the most important previous models for ranking efficient units cannot rank non-extreme ones. Additionally, in most previous studies, DEA models combined with PCA fail to handle negative data, while, the presented models can cover this data. Two case studies (pharmaceutical companies listed on the Iranian stock market and bank branches) are manipulated to demonstrate the applicability and performance of the algorithm. To show the superiority of the presented models, the SBM model without PCA and the Super SBM model without PCA have been implemented on the data of both cases. In comparing the two methods (PCA-SBM and SBM), the PCA-SBM model has higher ranking power (five efficient DMUs versus nineteen in the case of pharmaceutical companies and four efficient DMUs versus twenty-nine in the case of bank branches). Also in comparing the PCA-Super SBM and Super SBM, the PCA-Super SBM model works more powerfully in complete ranking. As the Super SBM model cannot rank non-extreme units unlike the PCA-Super SBM. Consequently, the presented algorithm works successfully in ranking the DMUs completely (inefficient, extreme, and non-extreme efficient) with low complexity.

## Introduction

Data Envelopment Analysis (DEA) is a nonparametric mathematical method for evaluating the relative efficiency of homogeneous decision-making units (DMUs) with multiple inputs and outputs. The efficiency of the method is reduced, when there are many internally correlated input/output indices, and when the number of DMUs is insufficient relative to the number of input/output indices.

In the DEA literature, there is a rule of thumb (Cooper [1]), the results of the implementation of the model can be accepted as long as the relation (1) applies.

Number of units evaluated > = Max ((number of inputs * number of outputs), 3 (number of inputs + number of outputs)) (Relation 1)

If relation (1) does not apply to the model, the number of efficient units increases, therefore, it becomes a challenge to distinguish and rank them. Similar inputs/outputs must be combined, or several must be deleted to solve this issue. For example, Toloo and Tichy [2] proposed a model for selecting specific inputs/outputs, where they extended both the multiplier and envelopment forms of DEA and suggested two alternative approaches, in order to select performance measures under Variable-Returns to Scales (VRS). Furthermore, Toloo et al. [3] developed various selected DEA models to restrict the performance factors, based on a rule of thumb. For solving this problem In this paper PCA, as a mathematical algorithm, was used to define the additional input/output indices, which linearly combine the preceding ones and are entirely independent of one another. Also, PCA can aid in reducing the number of inputs/outputs in DEA models. This technique can be used to define new indices (performance factors) by examining the data variance. The number of new indices is less than the number of the existing indices and their variance has the slightest decrease in comparison to the previous ones, resulting in minimal information loss.

The studies conducted in the field of combining both methods (PCA/ DEA) are divided into two groups:

- Those that employ the PCA and use its outputs as inputs of the DEA. These models are not able to provide any benchmarks or reference points for inefficient DMUs [4–15].
- Those that construct a model by combining both methods and entering data into a single integrated model so can give targets for inefficient DMUs to guide them to achieve full efficiency. The remarkable point about these researches is that they cannot handle negative data [16–22].

Our paper is classified in the second group and can provide the benchmarks and reference points. But unlike the studies in this group [16–22], our models have the ability to tolerate negative data. In some cases, the input/output data attain negative values (for instance gross profit-to-sales ratio (GPM)). As our proposed models are nonradial, in which the efficiency criteria are not sensitive to the data signature, they can handle negative data.

In order to use the PCA technique, it is required to add/subtract some values to the original data. These operations cause fundamental pitfalls in efficiency and ranking calculations in DEA models (Constant-Returns to Scales (CRS) models). As in this study, the VRS models are used and these models are translation-invariance so the presented models are stable in dealing with such modifications (addition/subtraction).

Also if the data of output indices is fractional, such as Net Profit Margin (NPM), the SBM models are not able to use them. Instead of using fractional data, researchers in this field believe that its numerator should be used as an output and its denominator as an input. This is feasible when both numerator and denominator are available but is not feasible when only fractional data is available. The model presented in this study begins by performing a series of changes to the data using PCA (there is nothing wrong with entering fractional data into PCA), which causes the data to exit their fractional mode. As a result, the model is also applicable to this data type while outperforming SBM models.

In this study, exploiting the PCA-SBM model results in a significant reduction in the number of efficient DMUs. Despite utilizing the PCA-SBM, there is a probability that the model selects more than one unit as being efficient. Ranking of efficient DMUs is conducted based on different methods. The most important approach is the super-efficiency method [23]. In this method, the efficient DMU (under evaluation) is removed from the set of observations (Production Possibility Set (PPS)) and then its efficiency is estimated. If this unit is non-extreme efficient, the new PPS is identical to the previous PPS. Therefore, there is no change in the calculation of efficiency and super-efficiency. This means that the super-efficiency method is incapable of distinguishing and ranking non-extreme efficient units. Though, unlike the fact, the super-efficiency method cannot rank the non-extreme efficient units, the PCA-SSBM (technique) is capable of ranking all units (extreme efficient, non-extreme efficient, and inefficient). Another imperative method for ranking the extreme and non-extreme efficient units is the cross-efficiency method. Given input-output data for a set of DMUs, a linear DEA program generates for each DMU, an efficiency score plus unit-specific weights or shadow prices for the entire inputs and outputs [24]. These weights can be used to fill a square matrix of so-called cross-efficiency values, where, each unit is appraised by each unit. Averaging those values row- or column-wise delivers aggregate measures for comparing the efficiencies of the production units. However, as is well known, the weights may not be unique, and therefore much of the discussion in the literature is concerned with as to how appropriate weights can be selected [24]. If the optimal weights are not unique, the cross-efficiency matrix will not be unique either. To solve this problem, various methods have been presented. In all these methods, various optimization problems are solved to find exclusive optimal weights. It results in a very high computational complexity. In this paper, the presented algorithm ranks the selected efficient units using a modified super-efficiency SBM, PCA-SSBM (presented in this paper). In the proposed method, computational complexity is reduced by solving a small number of optimization problems with the aid of the PCA procedure.

To show the applicability and performance of the algorithm, two case studies with different behaviors were selected: Pharmaceutical companies and branches of a bank: Pharmaceutical companies are one of the pillars of the health industry in the countries. Determining the efficiency of these companies and ranking them based on their efficiency will be useful for policymakers in this field. In this paper, by using the presented algorithm, twenty-seven pharmaceutical companies listed in the Iranian stock market were ranked completely based on financial efficiency. With the help of this information, the Ministry of Health can provide financial facilities (such as low-interest loans) to companies with better ratings and management advice to companies with worse ratings. And also with this information, stock market investors can select their stock portfolio. Ranking of a bank branches can help bank managers to determine appropriate rewards for the best and punishment for the worst. What is the fundamental difference between the two case studies, is explained in the Results and Discussions section.

### Innovations

- Ranking of DMUs while input/output data assumes positive/negative values.
- SBM development by utilizing the PCA technique to improve the ranking power of DEA by increasing the differentiation power among DMUs
- Utilization of PCA in super-efficiency SBM model to complete ranking of DMUs with low complexity.
- Combing PCA with DEA in order to reduce evaluation indices with the least loss of information.

This paper is structured as follows: Literature Review which begins with the definitions of two methods (DEA, PCA) and follows with three parts to review previous studies: The complete ranking of DMUs with negative input/output (as this paper focuses on negative data), DEA combined with multivariate techniques except PCA (as this paper focuses on the integrated method), and DEA with PCA (as this paper utilizes these two methods). Materials and Methods, in this section a combination of steps and models that are in both methods, as well as the proper order of the steps, has been mentioned. Moreover, it has been tried to make the presented algorithm understandable for the readers with sufficient explanations of the steps. A flowchart for the ranking algorithm. In this part, the steps of the algorithm are described in the form of a flowchart that can be seen at a glance, and in fact, the roadmap of the method is presented. Results and Discussions: the efficiency and ranking of bank branches and pharmaceutical companies which are calculated with the algorithm, are presented in this section. To express the superiority of the algorithm, the efficiency scores for two case studies also were calculated with SBM (Tone (2001)) [25] (without the PCA), and super-efficiency SBM (Tone2002) [23] (without the PCA). A comparison of the results and implementation times (between our models and SBM and SSBM models) is presented at the end of this section. Conclusions and Suggestions consist of the advantages and limitations of the selected approach and recommendations for future research.

## Literature review

Charnes et al. [26] proposed DEA as a nonparametric mathematical method for measuring the relative efficiency of homogeneous units with multiple input and output indices. This method could estimate the efficiency of the desired units by utilizing linear programming models and incorporating available data as the input/output of the model. According to Charnes et al., the proposed measure of a DMU’s efficiency is the maximum ratio of weighted output to weighted input which is subject to the condition of similar ratios for each DMU, which are less than or equal to one. In a more precise form:

$$\begin{array}{l} max\;h_{0}=\frac{\sum_{r=1}^{s}u_{r}y_{r0}}{\sum_{i=1}^{m}v_{i}x_{i0}}\\ s.t.:\\ \;\frac{\sum_{r=1}^{s}u_{r}y_{rj}}{\sum_{i=1}^{m}v_{i}x_{ij}}<=1;\;j=1,\ldots \ldots n,\\ u_{r},v_{i}>=0;\;r=1,\ldots .s;\;i=1,\ldots .,m. \end{array}$$ (1)

Where y_rj_ and x_ij_ (all positive) denote the known output and input of the *j*_th_ DMU.

The Principal Component Analysis (PCA) is one of the multivariate techniques which concerned with explaining the variance-covariance structure of a set of variables through the use of several linear combinations of these variables, with the overall objective being data reduction and interpretation. As stated by Johnson and Wichern [27], let us assume that the random vector X [X_1,_…., X_p_] (the original input/output to be aggregated) has the covariance matrix V with eigenvalues λ_1_ > = λ_2_> =… ..> = λ_p_> = 0 and eigenvectors l_1_,l_2_,…., l_p_. Consider the linear combinations as principal components (PCs): (the transpose operator denoted by the superscript t)

$$\begin{array}{l} X_{PC_{i}}=l_{i}^{t}X=l_{1i}X_{1}+l_{2i}X_{2}+\ldots .+l_{pi}X_{P},\\ Var(X_{PC_{i}})=l_{i}^{t}Vl_{i},\;i=1,2,3,\ldots ,p\\ Cov(X_{PC_{i}},X_{PC_{k}})=l_{i}^{t}Vl_{k}\;i=1,2,3,\ldots .,p\;k=1,2,3,\ldots ..,p \end{array}$$ (2)

The PCs are uncorrelated linear combinations X_pc1,_ X_PC2, …._ ranked descendingly by their variances.

As in this study, the combination of PCA and DEA is used to complete rank of DMUs with negative data, previous studies on these subjects are reviewed:

### Studies on the complete ranking of DMUs with negative input/output

Studies in this field can generate complete ranking in the presence of negative data by using the super-efficiency approach or other approaches. Hadi-Vencheh et al. [28] defined a new super-efficiency model in the presence of negative indices (input/output). This model ranked all DMUs using the Range Directional Model (RDM) and determined the efficiency type of the DMUs, but in this model, sometimes infeasibilities exist which is the weakness of the model. Lin et al. proposed [29] a DDF-based (Dynamic Data Flow) super-efficiency model to complete rank of units with positive and/or negative data by applying the directional vectors. This model is sensitive to the value of parameter K which influences the power of ranking of the method. Babazadeh et al. [30] proposed a novel radial (DDF-based) super-efficiency model to distinguish between efficient and inefficient units. Furthermore, this model could generate a complete ranking order for all DMUs using a new super-efficiency measure. Additionally, it ensured feasibility regardless of whether the input/output data were positive or negative. Moreover, the given model possessed advantages such as monotonicity, unit invariance, and translation invariance properties. Some studies based on other approaches can be stated as follows: Wei et al. [31] introduced a novel modified SBM (Slacks Based Measure) that allowed for negative input/output values and ranked all the DMUs. This novel model was defined as a unit- and translation-invariant, two critical properties of DEA models. Additionally, they proposed this method to assist decision-makers in evaluating the performance of all units and assigning them a complete ranking in the presence of negative data. Lin [29] defined the VRS cross-efficiency evaluation for DMUs as a ratio, capable of handling positive and negative data in the input and output. For all the DMUs, the resulting cross-efficiencies and scores were between zero and one, which ensued in their ranking. Of course, one of the major drawbacks in complete ranking with the cross-efficiency method is that the cross-efficiency matrix is not unique. Soltanifar et al. [32] have solved this problem by combining this method with the fuzzy VIKOR method.

### DEA with multivariate techniques (except PCA)

Researchers have used the combination of DEA with different techniques to improve the performance of the DEA method. Combining the DEA method with multivariate techniques (PCA, Factor Analysis, and Clustering,) is usually done for reducing input/output indicators or clustering input/output indicators. Clustering in DEA can help to solve the problem of heterogeneity in input and output data. Among the most important studies for integrating FA and DEA, the following can be stated: Nadimi and Jolai [33] combined DEA and FA for data reduction in DMUs. Their numerical results indicated that the new approach was highly consistent with DEA in terms of ranking. Vargas and Bricker [34] combined the CCR (Charnes, Cooper, and Rhodes) output-oriented model of DEA and FA to evaluate the academic units of several graduate programs with their national counterparts. They acknowledged that many academic program outputs are not immediately visible. Thus, the FA separated these outputs from the visible output and then evaluated the units using these outputs by DEA. Huang and Chen [35] developed a novel FA-DEA method based on classification and weighting, resulting in an improved model based on the classification of the original indices. Gong, Shao, and Zhu [36] proposed a new method for evaluating the energy efficiency of ethylene production by incorporating DEA and FA into the operation classification. Mengdie Huang et al. [37] conducted their evaluation employing a multi-attribute efficiency evaluation model, a multivariate statistical method, and grey theory, a so-called DEA integrated grey FA (GFA-DEA) approach, to support efficiency evaluation and ranking in uncertain systems. In this study, an efficiency measures generation technique is integrated into the grey factor analysis method for the benchmarking of DMUs. Among the most important studies for integrating Clustering and DEA, the following can be stated: Azadeh et al. [38] demonstrated how to optimize operator allocation in Cellular Manufacturing Systems using integrated fuzzy DEA (FDEA), fuzzy clustering C-means (FCM), and a computer-based simulation (CMSs). This study included integrated simulation, FDEA, FCM, and fuzzy indices, Mike G. Tsionas [39] combined Clustering and meta-frontier in DEA for solving the problem of heterogeneity of DMUs. He provides a prior design to minimize variation within groups and maximize variation across groups. The techniques are applied to a data set of large U.S. banks.

Of course, multivariate techniques are not the only way to reduce indicators in DEA, for example, Xi Bao et al [40] focused on a combination of fuzzy (QFD) and interval DEA to provide a new two-phase methodology for DEA indicator screening. The study is the first attempt to overcome the curse of the dimensionality problem and screen the evaluation indicators by combining fuzzy QFD and interval DEA.

In our paper, the enhanced models which combine PCA and SBM, PCA and Super SBM to improve the ranking power of the DEA method are proposed.

### DEA with PCA

The studies that combined both methods (namely, PCA and DEA) are divided into two categories: Those that employ one method and use its output as input for another method (i) (which cannot provide any benchmark for inefficient units), and those that construct a model by combining both methods and insert data into the integrated model (ii) (which can provide benchmarking). It should be noted that this paper is classified as the second category.

- Azadeh et al. [4] used a combination of two PCA-DEA methods to compare the wireless communication sectors of forty-two countries. The first step was to use the PCA method to convert seventeen indices into three input and five output indices. The second step was to rank countries according to DEA. Azadeh et al. [5] developed an integrated approach for assessing and optimizing total energy efficiency in energy-intensive manufacturing sectors using DEA, PCA, and Numerical Taxonomy (NT). Additionally, they presented a comprehensive approach that included structural and conventional energy efficiency indices, verification and validation mechanisms for DEA via PCA and NT; and the use of DEA for assessing total energy efficiency and optimizing consumption in energy-intensive manufacturing sectors. Wu et al. [6] connected the PCA and DEA methods to estimate online banking performance (giant banks in the USA and UK, including financial and nonfinancial variables). Amirteimoori et al. [7] proposed a method for estimating the efficiency of DMUs through PCA. This method allows for handling undesirable output while reducing the dimension of the data sets. They accomplished this by first reversing the undesirable output. Then used PCA to calculate the ratio of a single desired output to a single input. Finally, the selected PCs were imported into DEA as virtual data sets. The proposed approach was validated using a data set of the Iranian bank branches. Chunyan Yao [8] used a PCA-DEA model to determine the level of regional innovation in thirty Chinese provinces and cities and converted the correlated input/output variables to uncorrelated ones. Simultaneously, a smaller number of primary components was identified to express the original data. Finally, the DEA model was constructed using uncorrelated variables. Azadeh et al. [9] used DEA and PCA to evaluate the current maintenance and Health, Safety, and Environment (HSE) management systems of a gas transmission unit. Furthermore, they presented an integrated HSE and a performance-optimization system maintenance strategy. Moreover, PCA was used to verify the results. Poldaru and Roots [10] assessed the Quality Of Life (QOL) scores in Estonian counties using a PCA-DEA model and analyzed the model’s results. The procedure involved a two-stage analysis that began with a PCA. The standard DEA was used in the second stage. Zha et al. [11] applied PCA to analyze the original value judgment information, and the key indices in the production process are extracted. The modified DEA models are proposed. DEA efficiencies and their projections are calculated. Faed et al. [12] presented an intelligent system for handling customer complaints via PCA and DEA. They used PCA to analyze the data because it was more effective than other tools such as Exploratory Factor Analysis (EFA) in compressing data and producing more accurate results. The DEA was then used to differentiate critical customers from others and ascertain the most influential perspectives on policymaking. Omrani et al. [13] calculated the development degrees of provinces in various classes using the Common-weight DEA (CWDEA) model, the scores of which were used as PCA model indices. Additionally, they ranked the provinces using the PCA model. Xiao Shi et al. [14] evaluated the technical efficiencies of risk management of Chinese commercial banks by the DEA-BCC model. The PCA was applied to delete redundant input indicators. Rahimpour et al. [15] proposed a model to evaluate the performance of organizational units considering intellectual capital (IC) and the employee loyalty approach by applying the PCA-DEA method.
- Some previous studies that developed a model by combining both methods (PCA and DEA) and entering data into the integrated model are summarized in Table 1. In this Table the previously proposed models are compared with the model proposed in this paper in several ways: (I) supporting both positive and negative data; (II) using CRS/VRS models; (III) utilizing Radial/Nonradial models.

**Table 1:** Comparison of the studies with a model combining (PCA, DEA).

Publication	Subject	Support both Negative and positive data	Support Only positive data	CRS model	VRS model	Radial model	Nonradial model
Adler and Golany [16]	Evaluation of deregulated airline networks using DEA with PCA with an application to Western Europe.		√	√			√
Adler and Golany [17]	Including principal component weights to improve discrimination in data envelopment analysis.		√	√			√
Adler and Friedman [18]	Review of ranking methods in the data envelopment analysis context.		√	√		√	
Adler and Golany [19]	PCA-DEA Reducing the curse of dimensionality.		√		√	√	
Adler and Yazhemsky [20]	Improving discrimination in data envelopment analysis: PCA–DEA or variable reduction.		√	√			√
Andrejić et al [21]	Benchmarking distribution centers using PCA and DEA: A case study of Serbia.		√		√	√	
Jothimani et al [22]	A PCA-DEA Framework for Stock Selection in Indian Stock Market.		√	√			√
This paper (PCA-SBM)	Complete ranking of DMUs dealing with negative data using DEA and PCA	√			√		√

The primary advantage of the model of this research is that it is nonradial and defined on VRS, which means that the efficiency criterion is unrelated to the data signature. This advantage has not been documented in previous research to the best of the authors’ knowledge.

## Materials and methods

This part consists of two parts: explaining the steps of the methodology, and the roadmap (flowchart) of the algorithm implementation.

### Ranking algorithm steps (explanation)

Steps of the algorithm include two parts, steps 1 to 5 are the explanations of the PCA technique, and steps 6 and 7 are the explanations of the DEA models.

#### 1- Modifying data

The raw data modification was fulfilled in two steps as follows:

*1-1- Modifying input data*. When the values of input data for a unit are considerably low, the efficiency calculated by DEA is overestimated. Since efficiency is calculated by the ratio of output to input, the DEA categorizes an inefficient unit erroneously, as an efficient one. The high-efficiency score of this unit is not because of its appropriate operation, but is due to abnormally low values of some of the inputs; the algorithm presented in this paper has solved this problem.

To solve this issue, a penalty should be added to these data values. If the values are less than $\bar{X}-3\sigma$, a penalty equal to 2 ($\left(\bar{X}-3\sigma )-X)\right)$ will be applied. This step is taken to ensure that no DMU receives a high-efficiency score because of its low input data values (The Decision Maker (DM) determines the severity of the penalty).

*1-2- Modifying input/output data by subtracting data from the mean*. PCA can be performed on mean-corrected data or standardized data (mean-corrected data divided by standard deviation) [41]. When mean-corrected data are used, the variables’ relative variance affects the weights used to form PCs (Principal Components). As a result, the variable with the highest variance compared to the others will be given a higher weight, and vice versa. Standardized data can be used to avoid the effect of relative variance on the weights.

In DEA, when an index’s data dispersion is large, it should be more influential in creating new indices, so it is better to use mean-corrected data.

Mean corrected (*X*) = *X*-mean (*X*) Mean corrected (*Y*) = *Y*-mean (*Y*)

**Note 1:** Sometimes, mean-corrected data becomes negative, so it is necessary to use DEA models that are stable in data changes such as subtraction and to cover negative data.

**Note 2:** The modified data are denoted as *x* and *y*.

#### 2- Determining the variance-covariance matrix of the modified data

If the model’s input is denoted by *x*_*ij*_ (the value of the i-*th* input index for the j-*th* DMU), then the input variables’ variance-covariance matrix will be as follows: *(i*: *1 … m) (j*: *1 … n)*

$$A^{\prime }=(\begin{array}{ccc} \mathrm{var}(x_{1}) & \mathrm{cov}(x_{1},x_{2})\ldots & \mathrm{cov}(x_{1},x_{m})\\ \vdots & \ddots & \vdots \\ \mathrm{cov}(x_{m},x_{1}) & \cdots & \mathrm{var}(x_{m}) \end{array})$$ (3)

If the model’s output is represented by y_rj_ (the value of the r-*th* output index for the j-*th* DMU), then the output variables’ variance-covariance matrix will be as follows: *(r*: *1 … s) (j*: *1… n)*

$$A=(\begin{array}{ccc} \mathrm{var}(y_{1}) & \mathrm{cov}(y_{1},y_{2})\ldots & \mathrm{cov}(y_{1},y_{s})\\ \vdots & \ddots & \vdots \\ \mathrm{cov}(y_{s},y_{1}) & \cdots & \mathrm{var}(y_{s}) \end{array})$$ (4)

#### 3- Calculating eigenvalues and eigenvectors of the variance-covariance matrix

After determining the *A* and *A΄* matrixes, the eigenvalues and eigenvectors of these matrixes are calculated. The eigenvalues of *A΄* and *A* are denoted by *L՜*_*(1… m)*_ and *L*_*(1…s)*_. The eigenvectors of *A΄* and *A* are denoted by *e՜*_*(1…m) (1…m)*_ and *e*_*(1…s) (1…s)*_.

**Note:** The following equation is used to determine the eigenvalues of a matrix:

|A−LI|=0 (5)

Where *A* denotes the desired matrix, L represents the eigenvalues, and I is the unit matrix.

The eigenvalues are obtained by setting this determinant to zero. Then they are sorted in descending order, and the largest one is selected. The following equation yields the eigenvector corresponding to the chosen eigenvalue:

Ae−Le=0 (6)

Where *A* denotes the desired matrix, *L* denotes the eigenvalue, and *e* represents the eigenvector associated with this eigenvalue. Similarly, successive eigenvalues are used if necessary.

#### 4- Calculating new variables (PCs)

The new variables are thus defined as follows:

$$\begin{array}{l} y_{rj}^{PC}=\sum_{r=1}^{s}e_{qr}y_{rj},\;x_{ij}^{PC}=\sum_{i=1}^{m}e_{hi}^{\prime }x_{ij} \end{array}$$ (7)

*x*_*ij*_: Modified value of i-*th* old input variable (index) for j-*th* DMU
*y*_*rj*_: Modified value of r-*th* old output variable (index) for j-*th* DMU
*q*_(_1 … a), a: Number of new output variables (PCs)
*r* (1 … s), s: Number of old output variables
*j* (1 … n), n: Number of DMUs
*h* (1 … f), f: Number of new input variables (PCs)
*i* (1 … m), m: Number of old input variables
*a*≤*s*,*f*≤*m*

**Note:** In Eq (6), for a unique eigenvalue (*L*), there is not a unique answer for the eigenvector (*e*). If any vector holds in this equation, its negative also holds. For example, if *e*^*(+)*^ is an eigenvector for an eigenvalue (*L*), *e*^*(-)*^ *= —e*^*(+)*^ is also an eigenvector for *(L)*. The key question is whether *e*^*(+)*^ or *e*^*(-)*^ and *e΄*^*(+)*^ or *e΄*^*(-)*^ should be used as an eigenvector in Eq (7). The concept of loading was used in this study to aid in selecting the correct eigenvector.

*Loading* indicates the extent to which the original variables *(x*_*ij*_,*y*_*rj*_*)* influence the new variables *(x*_*ij*_^*pc*^,*y*_*rj*_^*pc*^*)*. The higher the loading, the more influential the variables in creating the PC scores, and vice versa. The loading can be determined using the following equation for (x) [41]:

$$l_{hi}^{\prime }=\frac{e_{hi}^{\prime }}{S_{i}}\sqrt{L_{h}^{\prime }}$$ (8)

Where *l΄*_*hi*_ denotes the loading of the i-*th* input variable into the h-*th* input PC, *e΄*_*hi*_ (eigenvector) represents the weight of the i-*th* input variable in forming the h-*th* input PC, *L΄*_*h*_ represents the h-*th* input PC’s eigenvalue, and *S*_*i*_ denotes the i-*th* input variable’s SD (Standard Deviation).

Thus, the following equation for (y) can be used to determine the loading:

$$l_{qr}=\frac{e_{qr}}{S_{r}}\sqrt{L_{q}}$$ (9)

Where *l*_*qr*_ denotes the loading of the r-*th* output variable into the q-*th* output PC, *e*_*qr*_ (eigenvector) denotes the weight of the r-*th* output variable into forming the q-*th* output PC, *L*_*q*_ represents the q-*th* output PC’s eigenvalue, and *S*_*r*_ represents the r-*th* output variable’s SD.

For the Eq (7), the total loading (*TL΄)* value is further calculated as follows: (inputs)

$$TL_{h}^{\prime }=\sum_{i=1}^{m}l_{hi}^{\prime }$$ (10)

The *TL΄* values for *e΄*^*(+)*^ and *e΄*^*(-)*^ are estimated, and the one with the highest *TL΄* is selected as the eigenvector (*e΄*). Since *TL΄* indicates the effect of the old input variables (*x*) on the new ones (*x*^*PC*^), the greater the effect, the more desirable.

For the equatiotion (7), the total loading *(TL)* value is calculated as follows: (outputs)

$$TL_{q}=\sum_{r=1}^{s}l_{qr}$$ (11)

The *TL* values for *e*^*(+)*^ and *e*^*(-)*^ are estimated, and the one with the highest *TL* is selected as the eigenvector (*e*). As *TL* indicates the effect of the old input variables (*y*) on the new ones (*y*^*PC*^), the greater the effect, the more desirable.

#### 5- Determining the number of new input/output variables (PCs)

When the requirement arises to minimize the number of efficient units, only one input/output index should be taken into consideration. As a result, the values of *h* and *q* in the following equations must be equal to one (*h* = 1, *q* = 1). In this case, the percentage of the total variance of the original input data retained in the model is as follows:

$$R_{h}^{\prime }=\frac{L_{1}^{\prime }}{\sum_{i=1}^{m}\mathrm{var}(x_{i})}$$ (12)

Concerning the output data, the given percentage will be as follows:

$$R_{q}=\frac{L_{1}}{\sum_{r=1}^{s}\mathrm{var}(y_{r})}$$ (13)

The more the number of inputs (h > 1), the more the number of efficient units. Consequently, the percentage of the total variance of the original input data retained in the model will be as follows:

$$R_{h}^{\prime }=\frac{\sum_{h}L_{h}^{\prime }}{\sum_{i=1}^{m}\mathrm{var}(x_{i})}$$ (14)

The more the number of outputs (*q* > 1), the more the number of efficient units. Thus, the percentage of the total variance of the original output data retained in the model will be as follows:

$$R_{q}=\frac{\sum_{q}L_{q}}{\sum_{r=1}^{s}\mathrm{var}(y_{r})}$$ (15)

Similarly, when the number of inputs reaches *m* (*h* = *m*), and the number of outputs reaches *s* (*q* = *s*), Eqs (14) and (15) achieves one. Accordingly, the model retains 100% of the total variance of the original input/output data. However, this is undesirable because it increases the number of efficient units. If the number of inputs/outputs is greater than one, a higher percentage of the total data variance is used in the model. The number of efficient units increases, which is counter to the primary objective of the research; thus it seems to be a trade-off.

The amount of reduction in the number of inputs/outputs depends on the opinion of DM. The DM can provide an acceptable limit for the total variance of the retained data. On the other hand, as relation (1) is a hypothesis in this research, the number of inputs/outputs must hold in this relation.

#### 6- Implementing the PCA-SBM model

The following steps are used to create the model presented in this study:

6-1- The SBM model [25].


$$\begin{array}{l} Z=\underset{\lambda ,s^{-},s^{+}}{min}(\frac{1-(\frac{1}{m})\sum_{i=1}^{m}\left(\frac{s_{i}^{-}}{x_{ip}}\right)}{1+(\frac{1}{s})\sum_{r=1}^{s}\left(\frac{s_{r}^{+}}{y_{rp}}\right)})\\ s.t.:\\ \;\sum_{j=1}^{n}\lambda_{j}x_{ij}=x_{ip}-s_{i}^{-},\forall i\\ \;\sum_{j=1}^{n}\lambda_{j}y_{rj}=y_{rp}+s_{r}^{+},\forall r\\ \;\sum_{j=1}^{n}\lambda_{j}=1;\\ \lambda_{j}\geq 0;\forall j\\ s_{i}^{-}\geq 0;\forall i\\ s_{r}^{+}\geq 0;\forall r \end{array}$$ (16)

*x*_*ij*_: Value of i-*th* input variable (index) for j-*th* DMU
*y*_*rj*_: Value of r-*th* output variable (index) for j-*th* DMU
*x*_*ip*_: Value of i-*th* input variable (index) for the desired (p*-th*) DMU
*y*_*rp*_: Value of r-*th* output variable (index) for the desired (p*-th*) DMU
*r (1… s)*, s: Number of output variables
*j (1… n)*, n: Number of DMUs
*i (1…m)*, m: Number of input variables

*6-2- The RBM model*. As the *x*_*ip*_ and *y*_*rp*_ values must be positive, they can be replaced by $R_{i}^{-}$ and $R_{r}^{+}$, given the following values:

$$\begin{array}{l} R_{i}^{-}=\underset{}{Max}(x_{ij}:j=1,\ldots ,n)-\underset{}{Min}(x_{ij}:j=1,\ldots ,n),i=1,\ldots ,m\\ R_{r}^{+}=\underset{}{Max}(y_{rj}:j=1,\ldots ,n)-\underset{}{Min}(y_{rj}:j=1,\ldots ,n),r=1,\ldots ,s \end{array}$$ (17)

As a result, the model is modified as follows:

$$\begin{array}{l} Z=\underset{\lambda ,s^{-},s^{+}}{Min}(\frac{1-(\frac{1}{m})\sum_{i=1}^{m}\left(\frac{s_{i}^{-}}{R_{i}^{-}}\right)}{1+(\frac{1}{s})\sum_{r=1}^{s}\left(\frac{s_{r}^{+}}{R_{r}^{+}}\right)});\\ s.t.:\\ \;\sum_{j=1}^{n}\lambda_{j}x_{ij}=x_{ip}-s_{i}^{-},\forall i\\ \;\sum_{j=1}^{n}\lambda_{j}y_{rj}=y_{rp}+s_{r}^{+},\forall r\\ \;\sum_{j=1}^{n}\lambda_{j}=1;\\ \lambda_{j}\geq 0;\forall j\\ s_{i}^{-}\geq 0;\forall i\\ s_{r}^{+}\geq 0;\forall r \end{array}$$ (18)

*6-3- The RBM model converted into a linear model*. Assume *t* equals the following fraction:

$$\frac{1}{1+\frac{1}{s}\sum_{r=1}^{s}\frac{s_{r}^{+}}{R_{r}^{+}}}=t$$ (19)

So the model takes on the following form:

$$\begin{array}{l} Z=\underset{\lambda ,s^{-},s^{+}}{Min}(t-(\frac{1}{m})\sum_{i=1}^{m}\left(\frac{ts_{i}^{-}}{R_{i}^{-}}\right));\\ s.t.:\\ \;t+\frac{1}{s}\sum_{r=1}^{s}\frac{ts_{r}^{+}}{R_{r}^{+}}=1;\\ \;t\sum_{j=1}^{n}\lambda_{j}x_{ij}=tx_{ip}-ts_{i}^{-},\forall i\\ \;t\sum_{j=1}^{n}\lambda_{j}y_{rj}=ty_{rp}+ts_{r}^{+},\forall r\\ \;t\sum_{j=1}^{n}\lambda_{j}=1;\\ \lambda_{j}\geq 0;\forall j\\ s_{i}^{-}\geq 0;\forall i\\ s_{r}^{+}\geq 0;\forall r\\ t\geq \varepsilon ; \end{array}$$ (20)

Thus, the DEA model used in this study is obtained by substituting ${ts}_{i}^{-}$ with $s_{i}^{-},{ts}_{r}^{+}$ with $s_{r}^{+}$ and *tλ*_*j*_ with *λ*_*j*_ and also using Eq (17) for (*R+*, *R*^*-*^) and Eq (7) for the variables (*x*, *y*).


$$\begin{array}{l} Z=Min(t-\frac{1}{f}\sum_{h=1}^{f}\frac{s_{h}^{-}}{\underset{j}{\max}(\sum_{i=1}^{m}e_{hi}^{\prime }x_{ij})-\underset{j}{\min}(\sum_{i=1}^{m}e_{hi}^{\prime }x_{ij})});\\ s.t.:\\ \;t+\frac{1}{a}\sum_{q=1}^{a}\frac{s_{q}^{+}}{\underset{j}{\max}(\sum_{r=1}^{s}e_{qr}y_{rj})-\underset{j}{\min}(\sum_{r=1}^{s}e_{qr}y_{rj})}=1;\\ \;t\sum_{i=1}^{m}e_{hi}^{\prime }x_{ip}=\sum_{j=1}^{n}\lambda_{j}(\sum_{i=1}^{m}e_{hi}^{\prime }x_{ij})+s_{h}^{-},\;\forall h;\\ \;t\sum_{r=1}^{s}e_{qr}y_{rp}=\sum_{j=1}^{n}\lambda_{j}(\sum_{r=1}^{s}e_{qr}y_{rj})-s_{q}^{+},\;\forall q;\\ \lambda_{j}>=0;\;\forall j\\ s_{h}^{-}>=0;\;\forall h\\ s_{q}^{+}>=0;\;\forall q\\ t>=\varepsilon ;\\ \sum_{j=1}^{n}\lambda_{j}=t \end{array}$$ (21)

*x*_*ij*_: Modified value of i-*th* old input variable (index) for j-*th* DMU
*y*_*rj*_: Modified value of r-th old output variable (index) for j-*th* DMU
*x*_*ip*_: Modified value of i-*th* input variable (index) for the desired (p*-th*) DMU
*y*_*rp*_: Modified value of r-*th* output variable (index) for the desired (p*-th*) DMU
*q*_*(*_*1… a)*, a: Number of new output variables (PCs)
*r (1 … s)*, s: Number of old output variables
*j (1 … n)*, n: Number of DMUs
*h (1 … f)*, f: Number of new input variables (PCs)
*i (1 … m)*, m: Number of old input variables
*a*≤*s*,*f*≤*m*
e eigenvector (*y*)
e՛ eigenvector (*x*)

#### 7- Complete ranking of DMUs by using the improved Super SBM (PCA-Super SBM)

The final step is to rank the DMUs based on their efficiency scores as determined by the model. Although using the PCA-SBM model results in a significant reduction in the number of efficient DMUs, the model probably selects more than one unit as efficient. In the next step, the selected efficient units are ranked by the Super SBM model [23] with several modifications. The PCA-SSBM model, proposed and used in this paper, is able to complete rank the DMUs with lower complexity.

The model can be described as follows:

$$\begin{array}{l} Z=Min(t+\frac{1}{f}\sum_{h=1}^{f}\frac{\left|s_{h}^{-}\right|}{\underset{j}{\max}(\sum_{i=1}^{m}e_{hi}^{\prime }x_{ij})-\underset{j}{\min}(\sum_{i=1}^{m}e_{hi}^{\prime }x_{ij})});\\ s.t.:\\ \;t-\frac{1}{a}\sum_{q=1}^{a}\frac{\left|s_{q}^{+}\right|}{\underset{j}{\max}(\sum_{r=1}^{s}e_{qr}y_{rj})-\underset{j}{\min}(\sum_{r=1}^{s}e_{qr}y_{rj})}=1;\\ \;t\sum_{i=1}^{m}e_{hi}^{\prime }x_{ip}=\sum_{\begin{array}{l} j=1\\ j\neq p \end{array}}^{n}\lambda_{j}(\sum_{i=1}^{m}e_{hi}^{\prime }x_{ij})-s_{h}^{-},\;\forall h;\\ \;t\sum_{r=1}^{s}e_{qr}y_{rp}=\sum_{\begin{array}{l} j=1\\ j\neq p \end{array}}^{n}\lambda_{j}(\sum_{r=1}^{s}e_{qr}y_{rj})+s_{q}^{+},\;\forall q;\\ \lambda_{j}>=0;\;\forall j\\ s_{h}^{-}:free;\;\forall h\\ s_{q}^{+}:free;\;\forall q\\ t>=\varepsilon ;\\ \sum_{\begin{array}{l} j=1\\ j\neq p \end{array}}^{n}\lambda_{j}=t \end{array}$$ (22)

*x*_*ij*_: Modified value of i-*th* old input variable (index) for j-*th* DMU
*y*_*rj*_: Modified value of r-th old output variable (index) for j-*th* DMU
*x*_*ip*_: Modified value of i-*th* input variable (index) for the desired (p*-th*) DMU
*y*_*rp*_: Modified value of r-*th* output variable (index) for the desired (p*-th*) DMU
*q*_*(*_*1… a)*, a: Number of new output variables (PCs)
*r (1 … s)*, s: Number of old output variables
*j (1 … n)*, n: Number of DMUs
*h (1 … f)*, f: Number of new input variables (PCs)
*i (1 … m)*, m: Number of old input variables
*a*≤*s*,*f*≤*m*
e: eigenvector (*y*)
e՛: eigenvector (*x*)

### Ranking algorithm (flowchart)

**Fig 1:**
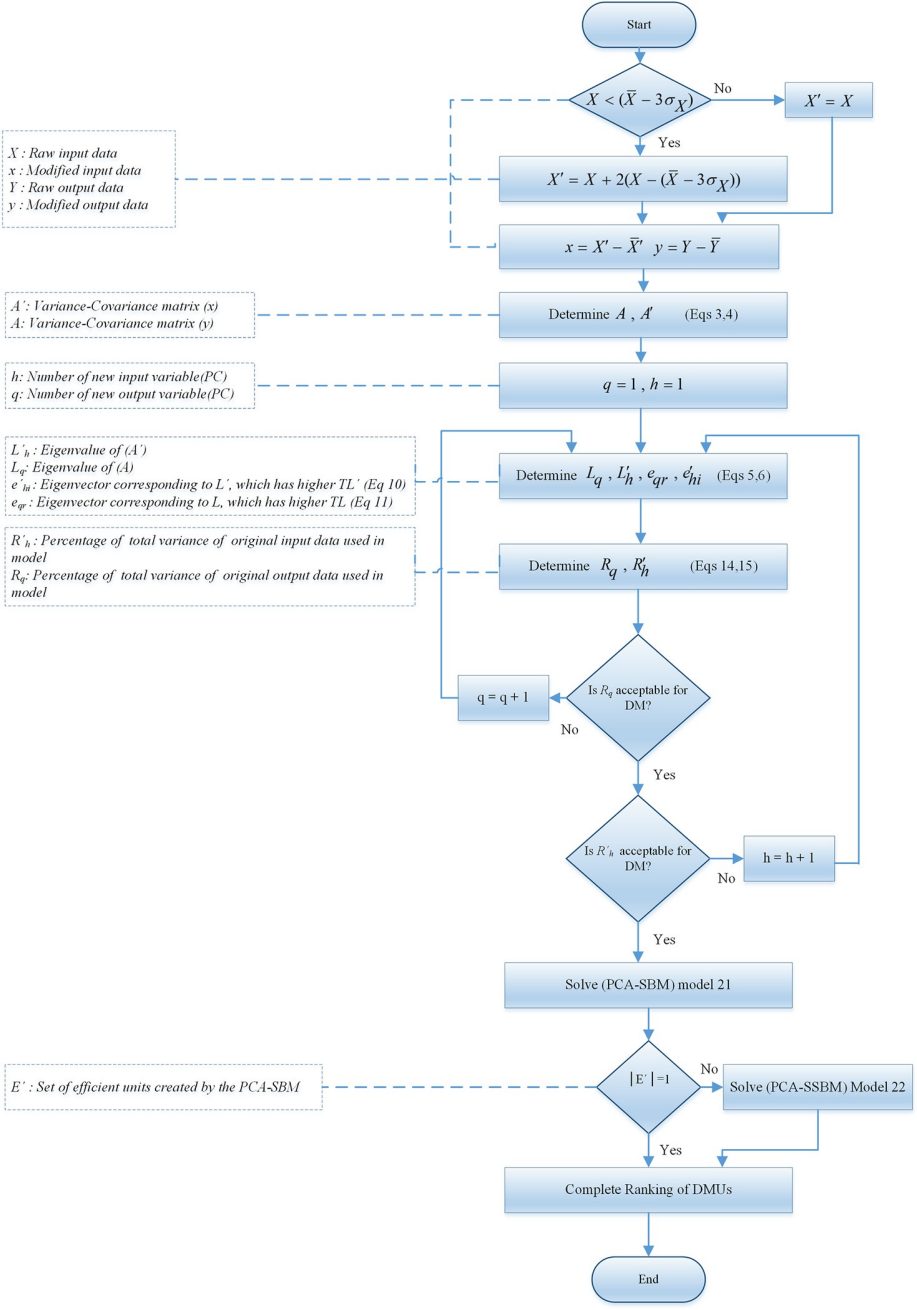
Ranking algorithm flowchart.

The steps necessary to implement the algorithm which are the roadmap of the method are depicted in Fig 1.

## Results and discussions

### Ranking the branches of a bank

In many papers, the DEA models are used to calculate the efficiency of banks. For example, Hatami-Marbini et al. [42] presented a method to assess the relative efficiencies of bank branches when interval and negative data were present. They classified bank branches as either strictly efficient, marginally efficient, or inefficient. Li et al. [43] estimated the efficiency of Chinese commercial banks by employing the super-efficiency DEA, Zhujia Yin et al [44] evaluated the banking efficiency by using the proposed DEA model and also Hssan Shah et al [45] investigated the efficiency, production technology gap, and productivity growth among 147 Central Banks (CBs) of South Asian countries from 2013–2018. Starting with financial indicators of CBs, various techniques such as DEA (CCR, BCC), DEA meta-frontier and Malmquist productivity index (MPI) are employed for the empirical analysis. Saif Ullah et al. [46] used the Data Envelopment Analysis (DEA) approach to measure the efficiency of the banks of Pakistan based on output and input variables. The study used data from seventeen commercial banks from 2011 to 2020 and also Hassan Shah et al. [47] employed the DEA Super-SBM with the undesirable output for the efficiency evaluation of 24 Central Banks of Pakistan. They were evaluated for 12 years, ranging from 2006 to 2017.

In our paper, the presented algorithm was applied to the data of forty bank branches in 2022. The data in this case study were initially positive, but some of them became negative by applying the PCA technique. The algorithm was used to estimate the efficiency scores of the branches, and then they were ranked.

Three input and eight output indices were used in this case study. To select indicators, first the available data were examined. Empirically, this fact can be used to distinguish between input and output indicators. The lower the input index value, the higher the efficiency of the banking system and the higher the output index value, the higher the efficiency of the banking system. The input indices include *personnel score (privilege of staff)*, *interests paid*, and *overdue receivables*. The output indices comprise *facilities*, *four deposits*, *interests received*, *fees*, and *other resources*.

The indices are described as follows (except for *personnel score*, the units of measurement for the given indices are in million Rials (Iranian currency)):

*Personnel score (privilege of staff)*: Refers to an index obtained from the number of personnel, their experiences, and their level of education and training. The index is calculated from the total weighted sub-index.

*Interest paid*: Banks pay interest on customer deposits. The total interest paid per month on all short- and long-term deposits is referred to as *interest paid*.

*Overdue receivables*: Lending money to customers is one of the bank’s activities. After receiving these loans, customers are required to repay them in monthly installments. Unfortunately, some customers do not pay them on time or at all. *Overdue receivables* refer to the amount owed by customers in installments.

*Facilities*: An index representing the value of the bank loans paid to customers. Markedly, this is the total amount of all facilities paid in one month.

*Four deposits*: Include interest-free, current, short- and long-term savings accounts held by customers. Notably, this is the balance of these deposits in a single month distinctively.

*Interests received*: Banks are always paid interest on facilities. The interests received are a percentage of loans. The sum of all interest received by banks for all previous loans, in a month, is referred to as the *interest received*.

*Fees*: Banks provide various services to their customers, including money transfers and guarantees, for which they charge a fee.

*Other sources*: The value of all other customer deposits, such as the government, is referred to as *other sources*.

**Note 1:** The number of new input indices/output indices to be extracted from the existing three inputs/ eight outputs is an important issue that depends on the opinion of the DM. Owing to the fewer the number of inputs/outputs, the lesser the number of efficient units, the higher the loss of information, so it seems to be a trade-off.

**Note 2:** Given that relation (1) is a research hypothesis, the number of inputs/outputs and the number of DMUs should apply in relation (1). When all three inputs and all eight outputs are considered, relation (1) holds: 40≥max{(3*(3+8)), (3*8)} [1]

The number of efficient units and the percentage of the variance of data used in the model were calculated for various inputs/outputs quantities, as expressed in Table 2.

**Table 2:** Number of efficient DMUs in bank branches, in different conditions.

Number of inputs	Number of outputs	Number of efficient DMUs	Percentage of variance of input data used in the model	Percentage of variance of output data used in the model	Relation (1)
1	1	4	99.424	80.333	√
1	2	7	99.424	98.380	√
2	1	11	100	80.333	√
2	2	12	100	98.380	√
3	3	20	100	99.278	√
3	8	29	100	100	√

- While the total number of primary indices (three inputs and eight outputs) are included in the model (the last row in the Table), thirty-one out of forty units become efficient, posing a significant challenge in ranking units, even though 100% of the variance of the input/output data is used.
- When the algorithm reduces the number of inputs and outputs to one (the first row in the Table), the number of efficient units decreases to four, which is significantly better than the previous condition for ranking units. The model retains 99.424% and 80.333% of the total variance of the input/output data, respectively. It is desirable if the DM is of the opinion that 80% of the variance of the input/output data is sufficient and it seems to be the best option for DM to accept because the number of efficient units is significantly less compared to other modes.
- When the number of inputs is reduced to two, 100% of the variance of the input data is utilized, indicating that, the three input indices are unnecessary. Thus, the model’s two independent inputs provide sufficient information.

**Fig 2:**
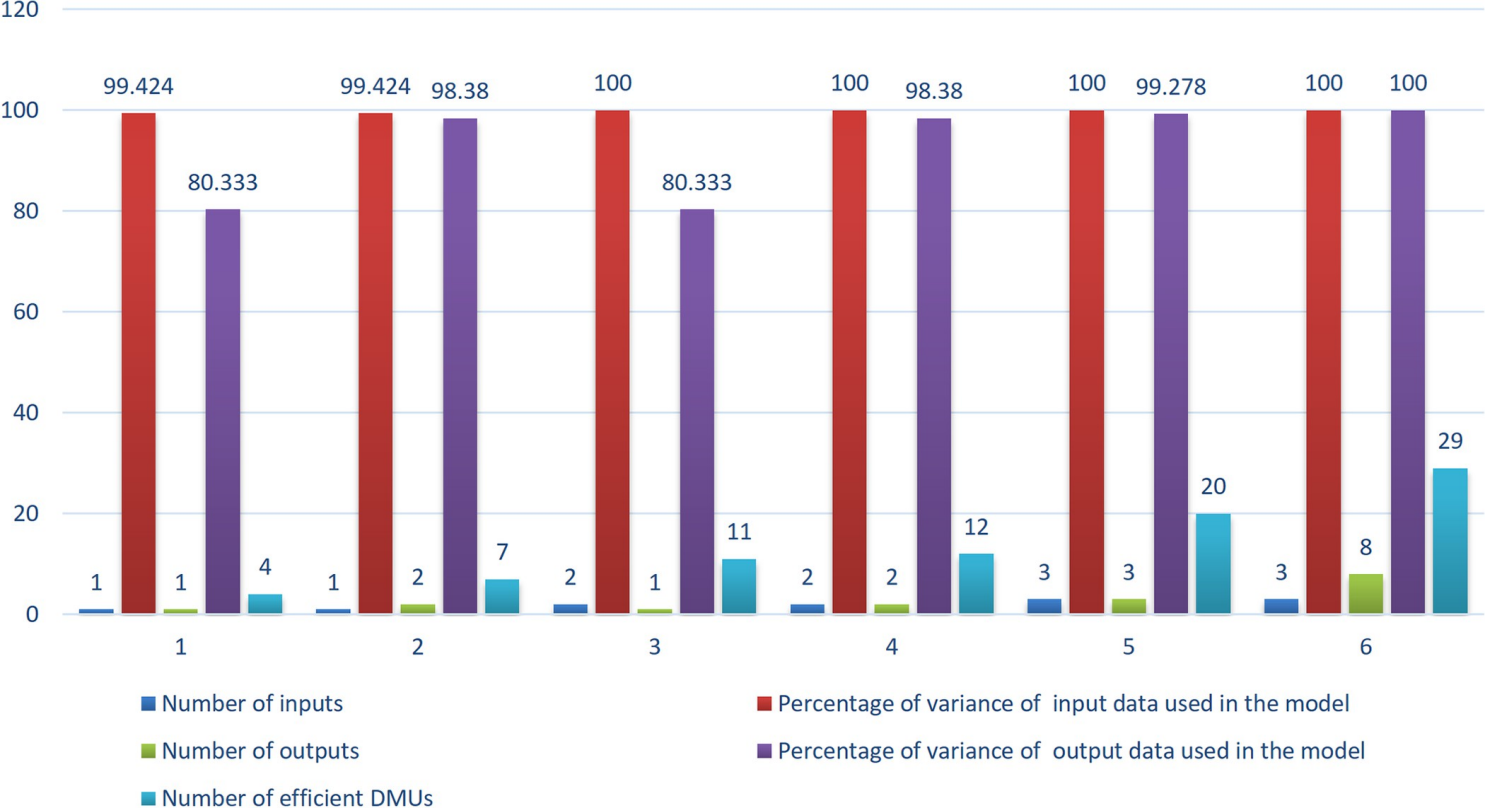
Comparing the number of inputs and outputs with the number of efficient units (bank branches).

As shown in Fig 2, the more the number of inputs and outputs, the more the number of efficient units, however the less the loss of information.

In Table 3, the bank branches are ranked according to their efficiency scores determined by the proposed algorithm for one input index and one output index. To express the superiority of this algorithm, the efficiency scores of the bank branches were calculated with SBM (Tone (2001)) [25] (without the PCA), and the score rank of the efficient units was estimated, using super-efficiency SBM (Tone2002) [23] (without the PCA).

**Table 3:** Ranking of bank branches (2022).

Branches	Efficiency score (PCA-SBM)	Score rank (PCA-SSBM)	Rank	Efficiency score (SBM)	Score rank (SSBM)
1	0.586785093		33	0.506860993	
2	0.766654173		25	1	1.008116943
3	0.805046267		17	1	1.061065397
4	0.971572699		6	1	1.023268292
5	1	1.002516586	4	1	1.218274724
6	0.381307054		37	0.405468219	
7	0.778294626		21	1	1
8	0.805739186		16	1	1.064652422
9	0.829099715		13	1	1.100104354
10	0.505932303		35	0.568165811	
11	0.768069056		24	1	1.089836236
12	0.724095227		27	1	1.022653912
13	0.695762369		29	1	1.073938377
14	0.723766177		28	1	1.078598548
15	0.771670576		23	1	1.021372534
16	0.967415441		7	1	1.363600886
17	0.380779277		38	0.359765349	
18	1	4.503120899	1	1	2.717937406
19	0.318681658		39	0.325985443	
20	0.864992326		10	1	1.048601566
21	0.538314119		34	0.622358974	
22	0.498321947		36	0.525649188	
23	0.755871701		26	1	1.094168317
24	0.695525023		30	0.652953933	
25	0.015830425		40	0.304000954	
26	0.613134194		32	0.574312787	
27	0.837343525		12	1	1.042189546
28	0.939194489		8	1	1.062259009
29	0.882076598		9	1	1
30	0.828257344		14	1	1.062477523
31	1	1.014254291	3	1	1.399656193
32	0.782016296		20	1	1.033054608
33	0.84956888		11	1	1.053931744
34	1	1.033492908	2	1	1.413353867
35	0.673816688		31	0.692425426	
36	0.776510752		22	1	1.020705561
37	0.790095951		19	1	1.085975872
38	0.802522841		18	1	1.031742471
39	0.975106941		5	1	1.033671314
40	0.81406494		15	1	1

We implemented our proposed method with GAMS software. In all cases, it was successful in calculating efficiency scores.

In comparing the two methods (PCA-SBM, SBM), it is clear that the PCA-SBM model works better than the SBM model (four efficient DMUs versus twenty-nine). If a method can assign the efficiency score of one to a smaller number of units, the ranking power of that method is higher. Because it is impossible to distinguish between units that have an efficiency of 1. the SBM method cannot capable to determine the rank for 29 DMUs: DMU2, DMU3, DMU4, DMU5, DMU7, DMU8, DMU9, DMU11, DMU12, DMU13, DMU14, DMU15, DMU16, DMU18, DMU20, DMU23, DMU27, DMU28, DMU29, DMU30, DMU31, DMU32, DMU33, DMU34, DMU36, DMU37, DMU38, DMU39 and DMU40. But the method PCA-SBM is not capable to determine the rank for only 4 DMUs: DMU5, DMU18, DMU31and DMU34.

When the two methods (PCA-SSBM, SSBM) are compared, it is distinct that the PCA-SSBM model works in a more enhanced manner than the SSBM model. Since the SSBM model cannot rank non-extreme units (DMU7, DMU29, and DMU40, as their efficiency scores remain one and it is impossible to distinguish between their ranks). Though, the PCA-SSBM is capable of ranking DMU7, DMU29, and DMU40 by assigning an efficiency score not equal to one to these units.

In S1 and S2 Tables in the Supporting Information section, raw data of input and output indices for the bank branches are presented (2022) [48].

### Ranking the pharmaceutical companies

Using the data available in the stock market to rank companies and select a portfolio via DEA has always been interesting for researchers. For example, Peykani et al. [49] proposed a robust two-phase approach for portfolio construction problem by using DEA and robust optimization approaches. The efficiency of all stocks that can be invested, was evaluated and measured using real-world data from Tehran Stock Exchange (March 2013 to March 2014).

Also using the DEA method for evaluating the efficiency of Health industry sectors such as hospitals and pharmaceutical factories is common. Anteneh Lamesgen et al [50]. was employed input-oriented DEA with a variable return to scale assumption to estimate the efficiency scores of neonatal health service and its associated factors among primary hospitals in three zones of Northwest Ethiopia. Wanhui Zheng et al. [51] utilized Four-Stage DEA to measure the relative efficiencies of Chinese public hospitals from 2010 to 2016. Rodrigo Moreno-Serra [52] applied DEA to analyze efficiency in the achievement of health system objectives and its relationship with certain system characteristics at the country level across Latin American and Caribbean (LAC) countries.

In this paper, the presented algorithm (PCA-DEA) was used to calculate the financial efficiency scores and rank twenty-seven pharmaceutical companies listed in the Iranian stock market (real-world data 2021). Ranking these companies can help stock market investors to select stock portfolios.

Some of the data in this case study was initially negative for example, *GPM* (Gross Profit Margin) and *ROA (*Return On Assets*)*, unlike the previous case study that there is no negative data between inputs and outputs of bank branches and after implementing the PCA technique, negative data is generated.

In this case study, three input indices, including *total investments*, *current assets*, and TFA (Tangible Fixed Assets), and eight output indices including *GPM*, OPM (Operating Profit Margin), *NPM* (Net Profit Margin), *ROA*, *ROE* (Return On Equity), *reverse P/E* (Price/Earning), *reverse P/B* (Price/Book Value), and *reverse P/S* (Price/Sales) were used. To select indicators, we have first examined the available data, the ones we need to perform manufacturing operations are the input indicators and the ones obtained after performing the manufacturing operations are the output indicators.

**Note 1:** For the last three indices (namely, *P/E*, *P/B*, *P/S*), the lower the values, demonstrate, the better organizational performance, hence, the inverse of these values are utilized for the output indices.

The indices are described as follows:

*Total investments*: Includes the total amount of initial capital as well as short- and long-term investments.

*Current assets*: Refers to cash and other assets that have been converted to cash or that have been sold and consumed during normal business operations within one year.

*TFA*: Generally refers to assets that have a physical value and are used by business units for their primary activities and are not excluded from any activity in the following fiscal year.

*GPM*: The company’s sales revenue after deducting direct production costs. *GPM* measures the gross profit-to-sales ratio; the higher this ratio, the more successful the business.

*OPM*: The company’s revenue after deducting expenses associated with its primary operations. The distinction between *GPM* and *OPM* is that operating costs are deducted in addition to production costs when estimating operating profit. Similarly, *OPM* calculates the operating profit-to-sales ratio; the higher this ratio, the more successful the business.

*NPM*: This type of profit is the residual income after all company expenses are deducted. The distinction between *NPM* and *OPM* is in nonoperational revenues and expenses. The most important items in this section are investment income, income from asset sales, financial expenses, and tax expenses. *NPM* calculates the net profit-to-sales ratio; the higher this ratio, the more successful the business.

*ROA*: Demonstrates a company’s management’s efficiency in utilizing its assets to generate earnings. *ROA* is also calculated by dividing the net profit by total assets; the higher this ratio, the more successful the business is.

*ROE*: Calculated by dividing net profit by total shareholders’ equity. Thus, *ROE* is regarded as a measure of a company’s profitability in terms of stockholders’ equity.

*P/E ratio*: This index is critical for stock valuation. It is calculated by dividing the market value per share (VPS) by the earnings per share (EPS). For calculating this ratio for a given year, the average stock price for the year is used as P, and the net profit for the year is divided by the number of common shares in the company as EPS; thus, the lower the index, the higher the value of the company’s shares; hence, the lower the index, the higher the value of the company’s shares.

*P/B ratio*: This index is calculated by dividing the company’s market value by its shareholders’ equity book value. This ratio is calculated in the desired year using the average stock price and total equity in the balance sheet of that year; thus, the lower the index, the higher the value of the company’s shares.

*P/S ratio*: This index is calculated by dividing the market value of the company by its sales. To calculate this ratio in a particular year, the average share price and the company’s sales from that year’s balance sheet are used; thus, the lower the index, the higher the value of the company’s shares.

**Note 2:** The number of new input/output indices to be extracted from the existing three inputs/ eight outputs is an important issue that depends on the opinion of DM. Because the fewer the number of inputs/outputs, the fewer the number of efficient units but the higher the loss of information, so it seems to be a trade-off.

**Note 3:** Given that relation (1) is a research hypothesis, the number of inputs/outputs and the number of DMUs should apply in relation (1). When all three inputs and all eight outputs are considered, relation (1) does not hold: 27≤max{(3*(3+8)), (3*8)}

[1] therefore, it is necessary to reduce the number of inputs and outputs.

The number of efficient units and the percentage of the variance of data used in the model were calculated for various inputs/outputs quantities, as expressed in Table 4.

**Table 4:** Number of efficient DMUs in companies, in different conditions.

Number of inputs	Number of outputs	Number of efficient DMUs	percentage of variance of input data retained in the model	percentage of variance of output data retained in the model	Relation (1)
1	1	4	81.963	82.886	√
1	2	5	81.963	92.801	√
2	1	12	97.053	82.886	√
2	2	14	97.053	92.801	√
3	3	17	100	98.251	√
3	8	19	100	100	˟

- While the total number of primary indices (three inputs and eight outputs) is included in the model (the last row in the Table), nineteen out of the twenty-seven units became efficient, posing a significant challenge in ranking the units, even though 100% of the variance of the input/output data is used. On the other hand, since the relation (1) is not established, this state is not accepted at all.
- When the algorithm reduces the number of inputs and outputs to one (the first row of the Table), the number of efficient units decreases to four and is significantly better than the prior situation for ranking units. The model maintains 81.963% and 82.886% of the total variance of the input/output data, respectively. It is desirable if the DM is certain that 80% of the variance of the input/output data is sufficient.
- While the number of inputs is reduced from three to one and the number of outputs is reduced from eight to two, the model retains 81.963% and 92.801% of the total variance of the input/output data, (the second row of the Table). As evident, the number of efficient units reached 5. As the number of efficient units has not much changed compared to the previous state, it seems to be the best option for DM to accept.
- While the number of inputs is reduced from three to two and the number of outputs is reduced from eight to two, the model retains 97.053% and 92.801% of the total variance of the input/output data, (the fourth row of the Table). As evident, the number of efficient units reached 14, reducing the ranking power of the model.

**Fig 3:**
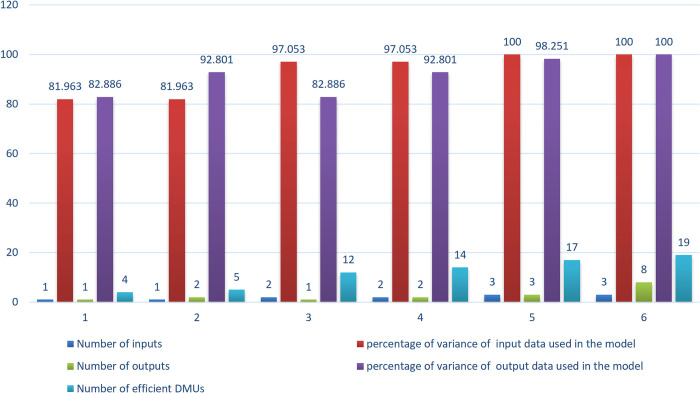
Comparing the number of inputs and outputs with the number of efficient units (pharmaceutical companies).

As shown in Fig 3, the more the number of inputs and outputs, the more the number of efficient units, however the less the loss of information.

In Table 5, the Pharmaceutical companies are ranked according to their efficiency scores determined by the proposed algorithm for one input index and two output indices. To express the superiority of this algorithm, the efficiency scores of the companies were calculated with SBM (without the PCA) [25], and the score rank of the efficient units was estimated using the super-efficiency SBM (without the PCA) [23].

**Table 5:** Ranking of the pharmaceutical companies (2021).

Company ID	Company Name	Efficiency score (PCA-SBM)	Score rank (PCA-SSBM)	Rank	Efficiency score (SBM)	Score rank (SSBM)
1	Jaber Ebne Hayyan Pharmaceutical Company	0.635446329		16	0.734495528	
2	Dr. Abidi Pharmaceuticals	0.034449716		26	0.233602535	
3	Iran Darou Pharmaceutical Company	0.916706236		7	1	1.165121402
4	Tehran Chemie Pharmaceuticals	0.17918436		25	0.793860448	
5	Darupakhsh Raw Materials Company	0.570726761		19	1	1.046884854
6	Amin Chemical and Pharmaceutical Co	1	1.056322314	4	1	1.500923365
7	Farabi Pharmaceutical Company	0.498106437		21	0.616304789	
8	Zagros Pharmed Pharmaceutical Company	1	1.003385894	5	1	1.470180503
9	Exir Pharmaceutical Co	0.716532905		14	1	1.144420551
10	Tehrandarou Co	0.502066059		20	1	1
11	Cosar Pharmaceutical Co	1	1.044530082	3	1	1.539153777
12	Chemi Darou Pharmaceutical Co	0.658916684		15	1	1.083259664
13	Zahravi Pharmaceutical Co	0.005036116		27	0.508587575	
14	Loghman Pharmaceutical hygienic Co	0.967255415		6	1	1.436053938
15	Rouzdarou Pharmaceutical Co	1	1.06582707	2	1	1.555196515
16	Shahid Ghazi Pharmaceutical Co.	0.803577885		9	1	1
17	Daropakhsh Pharmachem Chemical	0.737988919		13	1	1.097866564
18	Avicenna Laboratories Inc.	0.574141607		18	1	1
19	Aburaihan Pharmaceutical Co	0.746249621		11	1	1.02723783
20	Sinadarou Labs Company	0.841937335		8	1	1.16176448
21	Razak Pharmaceutical Co	Razak Pharmaceutical Co	0.462960392		22	0.689376971
Razak Pharmaceutical Co						
22	Daroupakhsh Pharmaceutical Co	0.788598589		10	1	1.290671261
23	Alborz Darou Pharmaceutical Co	0.322231206		24	0.542963906	
24	Pars Darou Pharmaceutical Co	0.590752976		17	1	1.078365426
25	Osvah Pharmaceutical Co	0.745491579		12	1	1.042343063
26	Sobhan Darou Pharmaceutical Co	0.448729266		23	0.678980381	
27	Iranian Parenteral and Pharmaceutical Company (I.P.P.C)	1	1.717854351	1	1	1.561950771

We implemented our proposed method with GAMS software. In all cases, it was successful in calculating efficiency scores.

Whilst comparing the two methods (PCA-SBM, SBM), it is evident that the PCA-SBM model works in a superior way than the SBM model (5 efficient DMUs versus 19). If a method can assign the efficiency score of one to a smaller number of units, the ranking power of that method is higher. Because the ranking of the units that get an efficiency score of one requires another complementary method. In this case study, the SBM method cannot capable to determine the rank for 18 DMUs: DMU3, DMU5, DMU6, DMU8, DMU9, DMU10, DMU11, DMU12, DMU14, DMU15, DMU16, DMU17, DMU18, DMU19, DMU20, DMU22, DMU24, DMU25 and DMU27. But the method PCA-SBM is not capable to determine the rank for only 4 DMUs: DMU5, DMU18, DMU31and DMU34.

When the two methods (PCA-SSBM, SSBM) are put to comparison, it is clear that the PCA-SSBM model works better than the SSBM model, because the SSBM cannot rank non-extreme units (DMU10, DMU16, and DMU18, as their efficiency scores remain one and it is impossible to distinguish between their ranks). Though, the PCA-SSBM is capable of ranking DMU10, DMU16, and DMU18 by assigning an efficiency score not equal to one to these units.

In S3 and S4 Tables in Supporting Information Section, raw data of input and output indices for the pharmaceutical companies are presented (2021) [53].

### Comparison of the complexity of two methods (SBM, Super SBM) with (PCA-SBM, PCA-Super SBM)

To compare the complexity of the proposed method with the complexity of the prior ranking method (SBM, Super SBM), the time of implementation (execution) can be applied as a complexity index. The less the time of implementation, the less the complexity.

The data set was different divisions of branches of one of the Iranian commercial banks. The maximum number of branches of this bank is around 2000. We have increased the number of DMUs exponentially in order to make the differences in execution times clear.

We implemented two methods with GAMS software, and applied several data sets from 50 to 2000 DMUs. In all cases, the software was successful in calculating efficiency scores. In SBM, Super SBM method, all indices (3 inputs and 8 outputs) are considered. The first step is to calculate the efficiency score of 50 DMUs with SBM and note down the time of its execution, the second step is to calculate the score rank of efficient units out of 50 with Super SBM and also note down the time, and finally add up these times. In PCA-SBM, PCA-Super SBM method only one input and one output are considered (which are actually a linear combination of the previous 3 inputs and 8 outputs). The first step is to calculate the efficiency score of 50 DMUs with PCA-SBM and note down the time of its execution, second step is to calculate the score rank of efficient units out of 50 with PCA-Super SBM and also note down the time, and finally add up these times. This time calculation method is continued for 100, 500, 1000, and 2000 DMUs.

Table 6 shows the implementation time of two methods for various numbers of DMUs (50, 100, 500, 1000, and 2000).

**Table 6:** Comparison of two methods.

Number of DMUs	Time of execution (SBM, Super SBM) (3 inputs, 8 outputs) (seconds)	Time of execution (PCA-SBM, PCA-Super SBM) (one input, one output) (seconds)
50	5.906	4.054
100	11.875	7.719
500	63.246	39.89
1000	137.766	79.861
2000	319.935	177.922

The results in the table show that in all cases, especially in the high number of DMUs, the complexity of the presented method (PCA-SBM, PCA-Super SBM) is far less than the previous method (SBM, Super SBM). The reason for this issue can be expressed from two perspectives: First, when the number of indices is reduced, the complexity of the model is less and it takes less time to solve the model. Second, as in the presented method, fewer units are selected as efficient units, so less time is required to calculate rank scores and perform their ranking.

**Fig 4:**
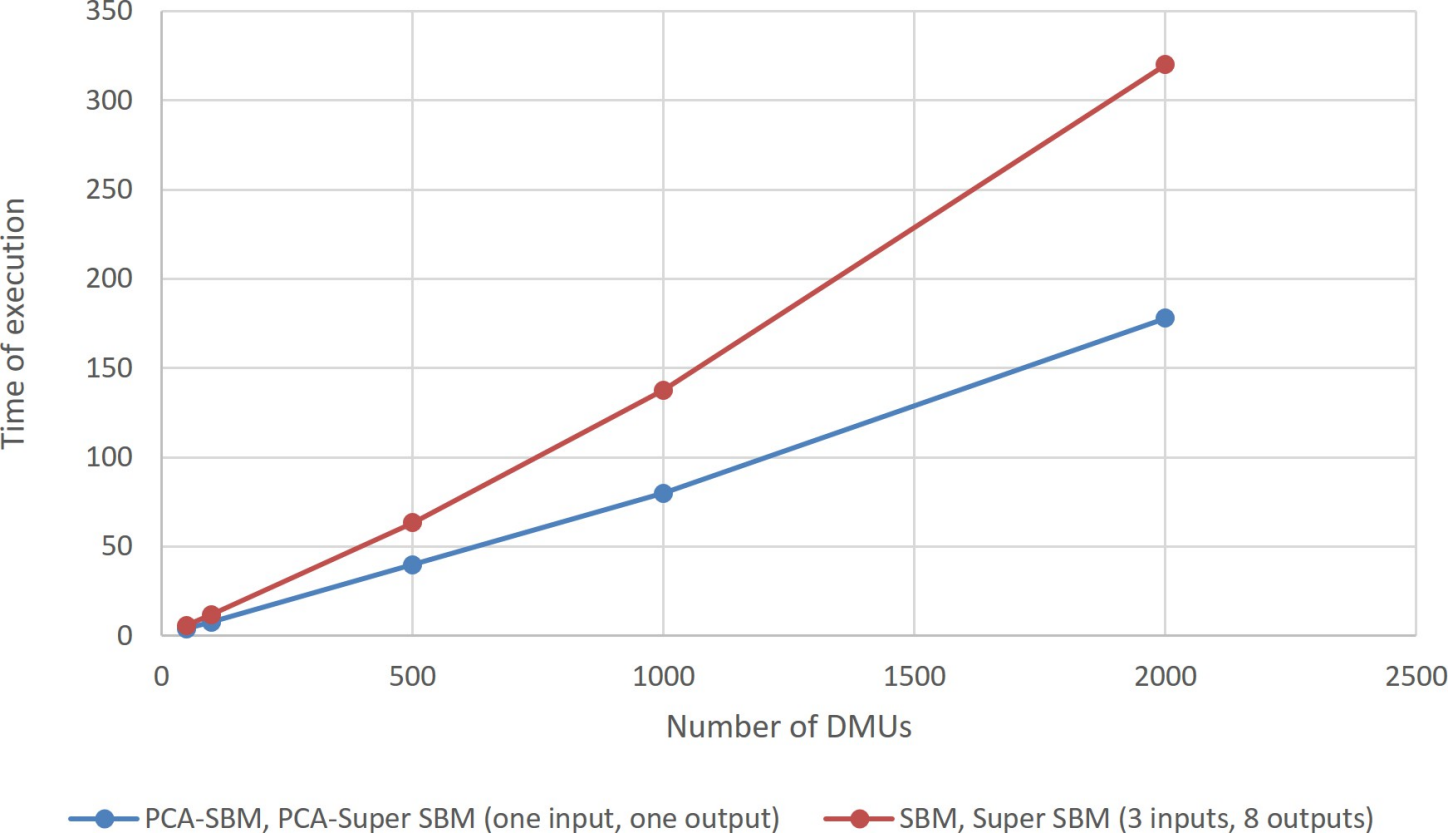
Complexity comparing.

In Fig 4 it is obvious that the complexity of the proposed method (PCA-SBM, PCA-Super SBM) is less than the complexity of the (SBM, Super SBM) method, especially in excessive amounts of DMUs.

## Conclusions and suggestions

In this study, the variable reduction concepts of the PCA technique were implemented in the SBM model and in the Super SBM model. The proposed models are feasible, stable, and computationally simple. Due to the SBM’s nonradial structure, slacks-based definition, and VRS definitions, the model is unit invariant, translation invariant, and covers both positive and negative data.

DMUs could be ranked completely with low complexity, by utilizing the algorithm presented in this paper. This claim was validated through the implementation of the algorithm in two case studies. An attempt has been made to implement the presented method for two sets of real data with different behaviors. The first category of data is related to bank branches, in which there is no negative data between inputs and outputs, but after implementing the PCA technique, negative data is generated and the appropriate DEA model is implemented on this data. The second category of data is related to pharmaceutical companies, where there is negative data among its inputs and outputs, and the presented model was also suitable for these data. By running the algorithm on both case studies that have different behaviors, we have checked its efficiency. In both cases (ranking the bank branches and ranking the Pharmaceutical companies), when the model PCA-SBM is used, the number of efficient DMUs decreases (compared to using SBM without PCA). This shows that the PCA-SBM model is more powerful in ranking DMUs compared to the SBM model. The PCA-Super SBM model (presented in this paper) also is able to rank all the DMUs (inefficient, extreme efficient, non-extreme efficient) in two case studies (compared to using Super-SBM without PCA which cannot rank non-extreme ones). To prove the low complexity of the presented model, also a comparison has been made between two methods (SBM, SSBM and PCA-SBM, PCA-SSBM) in terms of execution time for different numbers of units. By using this comparison, the low complexity of the presented method was proven especially in excessive amounts of DMUs.

Despite all the advantages of the presented method, there are also limitations in employing this method. First of all, the variance should be the main characteristic of the data and if the data of the input/output does not have the necessary correlation and variance to draw their correlation matrix with significant values, the PCA technique cannot be used for them. Additionally, to implement DEA, the units must be homogeneous and similar, and the input and output values must be independent of time.

Also If the input or output indices are qualitative, this algorithm cannot calculate the efficiency, so it is necessary to create models that combine two methods for fuzzy data, which can be addressed in future studies.

In the future, studies using the PCA-SBM model can provide any benchmark or reference points for the inefficient DMUs in order to improve their efficiency. In the introduced method, a single hybrid model exists, which can assist researchers to determine the amount of inputs and outputs modifications, so as to increase the efficiency of the inefficient units (whilst, in studies that manipulate the combination of the two techniques, (PCA and DEA) separately, this is impossible). After determining the amount of modifications in inputs and outputs of inefficient DMUs, the decision makers can define the policy for applying the modifications in order to convert inefficient DMUs to efficient ones. It is obvious that one of the factors affecting the policy can be the results of our paper.

The presented model will be developed to rank DMUs that have imprecise data (for example fuzzy data) in future studies. The important questions in forthcoming studies are “Which DEA models are suitable to be combined with the PCA, so as to cover imprecise data such as fuzzy data? “and correspondingly “If there is a network structure in DEA or if there are undesirable outputs, is it possible to combine DEA with PCA”?

## Supporting information

S1 Table Raw data of input indices for the bank branches (2022). (PDF)

S2 Table Raw data of output indices for the bank branches (2022). (PDF)

S3 Table Raw data of input indices for the pharmaceutical companies (2021). (PDF)

S4 Table Raw data of output indices for the pharmaceutical companies (2021). (PDF)
