# Supplementary material for: A novel algorithm for complete ranking of DMUs dealing with negative data using Data Envelopment Analysis and Principal Component Analysis: Pharmaceutical companies and another practical example
Source: PLoS One. 2023 Sep 1;18(9):e0290610. doi: 10.1371/journal.pone.0290610 (PMC10473491; doi:10.1371/journal.pone.0290610)
Supplement: S1 Table — (PDF) [file pone.0290610.s001.pdf]

S1 Table

Raw data of input indices for the bank branches (April 2022)

Branch ID	Personnel score	Interest paid (Million Rials)	Overdue receivables (Million Rials)
1	56.96	7,398,569,394	97,875,281,044
2	33.08	5,668,888,671	107,596,351,046
3	14.94	3,679,048,955	53,085,967,379
4	25.08	5,765,191,305	7,506,524,124
5	30.46	13,179,675,719	4,457,489,728
6	27.1	11,064,490,483	76,778,190,599
7	57.584	34,462,249,602	767,636,927,228
8	58.61	8,100,194,269	54,362,257,484
9	31.5	9,882,796,409	47,225,196,799
10	83.48	14,545,119,675	551,328,804,869
11	56.28	18,798,268,054	438,007,991,639
12	29.21	21,296,186,335	180,331,008,302
13	56.2	5,574,315,254	221,700,325,440
14	59.54	57,958,221,924	1,262,080,330,613
15	31.24	2,625,402,708	83,972,458,885
16	64.48	65,759,403,181	109,433,050,277
17	57.68	29,682,549,091	712,193,226,006
18	88.96	113,756,714,344	1,030,540,498,241
19	44.17	56,310,048,355	869,876,345,168
20	54.35	21,759,042,055	116,885,140,855
21	49.71	64,016,121,690	475,998,165,575
22	32.42	28,166,048,456	125,567,305,946
23	41.27	9,916,506,580	551,192,789,270
24	50.99	57,085,708,679	251,365,753,622
25	60.77	19,766,014,560	151,385,377,615
26	45.56	24,532,358,905	123,097,432,177
27	20.53	21,574,118,454	25,509,723,060
28	17.93	12,062,413,456	22,891,793,959
29	19.23	16,818,265,955	24,200,758,510
30	43.19	14,860,395,320	414,461,454,041
31	37.19	109,349,370,351	20,649,450,004
32	23.33	3,210,185,813	57,081,167,430
33	40.42	10,253,986,474	80,347,051,817
34	34.99	45,872,012,981	66,351,723,600
35	45.73	12,352,056,540	239,186,211,530
36	41.34	4,533,169,996	92,576,760,890
37	37.99	33,372,093,111	99,559,148,984
38	35.76	14,142,049,786	136,292,436,984
39	17.45	18,138,091,713	8,466,561,637
40	26.605	16,140,070,749	72,379,499,311
Mean	42.082725	26,085,685,384	245,785,848,292
SD	17.26330623	26,494,770,579	306,844,115,784
