# Supplementary material for: A novel algorithm for complete ranking of DMUs dealing with negative data using Data Envelopment Analysis and Principal Component Analysis: Pharmaceutical companies and another practical example
Source: PLoS One. 2023 Sep 1;18(9):e0290610. doi: 10.1371/journal.pone.0290610 (PMC10473491; doi:10.1371/journal.pone.0290610)
Supplement: S2 Table — (PDF) [file pone.0290610.s002.pdf]

S2 Table

Raw data of output indices for the bank branches (2022)

Branch ID	Facilities	Long-term savings	Current Savings	Interest-free savings	Short-term savings	Interest received	Fees	Other sources
1	108,530,545,234	16,268,693,708	44,051,681,827	3,769,112,259	22,509,681,176	949,531,431	430,128,957	4,164,785,534
2	130,254,342,064	11,909,298,282	54,070,676,218	2,416,261,612	11,879,098,444	771,820,528	953,243,936	13,359,943,686
3	52,594,293,366	8,033,792,000	79,314,137,769	4,065,986,848	11,481,738,611	31,873,575	622,028,381	2,839,966,859
4	55,829,566,545	11,309,508,694	40,047,909,353	4,570,023,724	16,775,461,606	99,827,652	667,990,551	8,535,467,418
5	93,948,996,571	27,300,770,918	15,377,868,956	4,386,291,425	26,878,124,242	618,835,874	301,983,987	2,287,407,864
6	39,932,392,725	29,778,690,000	98,794,997,616	3,201,550,458	12,032,469,266	717,708,953	69,194,508	1,364,706,195
7	443,240,309,799	93,135,508,568	117,258,676,134	8,414,967,735	35,247,587,292	9,645,262,273	2,363,675,616	36,603,085,736
8	123,030,721,197	21,690,560,393	19,797,439,290	6,346,168,820	8,658,447,337	3,143,682,788	246,855,352	2,353,418,509
9	156,904,389,033	13,615,845,888	57,018,354,861	13,847,707,932	33,664,491,841	814,683,845	655,421,038	14,634,649,003
10	305,925,067,013	34,704,364,645	50,251,237,565	1,788,618,284	22,139,531,333	2,515,286,144	1,131,060,735	5,731,467,426
11	260,407,697,433	48,390,089,173	156,624,915,000	2,181,661,348	41,650,876,874	5,484,766,122	3,192,804,333	42,022,469,114
12	181,013,589,656	53,054,882,000	13,767,115,204	945,465,423	57,796,814,401	7,614,794,562	49,267,019	2,422,289,524
13	144,645,113,262	12,835,463,690	44,582,636,336	933,583,285	10,036,223,321	1,091,440,058	983,263,853	20,617,374,000
14	717,489,228,347	160,253,637,661	58,209,317,835	17,764,927,317	25,642,652,918	15,886,006,501	1,119,982,540	28,474,010,668
15	39,861,147,337	4,677,474,291	84,885,730,222	1,493,688,473	21,558,718,920	27,736,559	113,715,977	1,523,625,703
16	1,136,643,015,436	122,670,485,687	34,499,927,009	2,080,848,519	154,774,073,447	37,914,989,912	655,170,254	8,634,302,610
17	82,503,298,087	55,837,534,905	19,424,878,825	919,641,428	77,656,404,853	449,280,091	47,728,568	932,845,079
18	1,788,095,774,021	263,934,217,656	2,195,604,272,680	11,769,230,349	235,145,496,366	3,446,932,857	28,391,765,445	30,778,620,587
19	616,439,183,679	63,734,708,051	52,592,182,939	1,438,940,378	206,410,176,918	10,118,798,933	920,253,366	10,953,027,261
20	314,249,753,565	55,420,624,250	486,055,043,835	1,566,947,521	18,254,687,685	401,155,342	660,902,666	8,386,442,803
21	131,528,943,012	212,698,966,419	68,120,857,507	1,469,960,283	38,593,644,472	1,002,813,109	586,723,617	14,924,378,171
22	147,718,119,825	40,099,324,016	28,737,702,152	2,542,521,093	99,482,189,752	737,164,161	355,447,282	3,283,480,264
23	79,542,213,812	25,596,339,600	219,927,958,597	2,342,990,189	11,229,760,336	769,155,317	416,682,981	6,924,721,486
24	180,355,089,085	166,432,258,674	131,631,740,413	2,032,710,629	36,605,012,323	353,159,943	209,301,677	7,749,213,040
25	44,348,441,580	47,965,156,517	31,470,282,946	2,685,946,953	27,367,837,172	2,444,886,640	927,963,021	12,166,745,991
26	547,719,743,672	41,257,663,228	49,887,224,839	1,723,776,793	38,843,613,982	1,496,477,997	1,115,154,658	6,719,207,235
27	28,883,698,009	81,741,090,000	25,973,713,324	1,365,342,506	36,033,461,350	203,129,667	246,216,888	3,262,162,552
28	419,493,867,209	34,661,050,000	73,961,181,695	2,212,958,363	12,206,616,255	438,356	616,993,538	16,227,503,177
29	224,188,782,609	58,201,070,000	49,967,447,510	1,789,150,434	24,120,038,803	101,784,011	431,605,213	9,744,832,864
30	379,496,680,114	17,927,720,000	117,560,504,970	3,864,169,710	55,387,554,951	9,834,423,601	311,826,933	2,487,980,504
31	736,499,780,180	228,222,026,000	21,527,893,294	1,162,250,260	142,107,977,200	29,872,403	21,021,185	154,588,000
32	20,565,250,221	7,644,243,438	1,134,341,006	1,282,219,193	8,263,653,186	209,308,411	35,200,599	590,525,474
33	447,054,737,337	6,173,992,273	113,668,145,375	1,317,975,030	61,193,845,634	3,495,563,167	303,110,775	4,606,417,515
34	1,165,621,601,965	126,269,780,000	10,414,762,066	3,799,548,337	13,656,564,253	962,851	37,680,653	3,261,236,746
35	88,904,058,277	32,674,862,372	18,426,135,626	1,465,195,069	9,816,565,236	1,906,771,718	649,457,246	11,452,896,932
36	160,492,101,841	10,403,144,354	24,996,122,183	2,627,042,493	3,562,702,987	2,492,449,204	191,953,090	3,136,964,889
37	241,228,440,006	43,409,973,248	36,910,521,141	5,019,200,834	181,892,750,872	193,521,795	405,871,262	627,135,484
38	242,234,496,932	66,144,198,408	279,564,255,531	2,468,083,476	25,961,646,073	3,792,305,834	495,856,711	1,299,173,961
39	137,244,413,903	51,183,413,109	14,137,500,608	2,909,673,224	31,247,923,456	1,483,949,261	138,887,607	8,233,582,366
40	189,739,455,417	58,663,805,759	146,850,878,070	2,688,878,350	28,604,784,765	2,638,127,548	317,372,159	4,766,378,164
Mean	310,109,958,484	61,648,155,697	129,677,454,208	3,516,780,409	48,409,272,498	3,373,266,975	1,284,769,104	9,205,975,760
SD	365,098,898,052	63,881,189,486	346,463,144,671	3,578,331,923	56,774,470,534	6,609,824,468	4,438,162,384	9,980,513,114
