# Supplementary material for: A novel algorithm for complete ranking of DMUs dealing with negative data using Data Envelopment Analysis and Principal Component Analysis: Pharmaceutical companies and another practical example
Source: PLoS One. 2023 Sep 1;18(9):e0290610. doi: 10.1371/journal.pone.0290610 (PMC10473491; doi:10.1371/journal.pone.0290610)
Supplement: S3 Table — (PDF) [file pone.0290610.s003.pdf]

S3 Table

Raw data of input indices for the pharmaceutical companies (2021)

Company ID	Total investments (Million Rials)	Current assets (Million Rials)	Tangible Fixed Assets (Million Rials)
1	3,227,347	9,420,125	1,703,083
2	4,786,396	23,874,627	13,197,517
3	1,153,681	4,590,183	125,628
4	1,203,267	7,197,184	797,265
5	1,252,853	9,804,184	1,468,901
6	4,367,842	8,256,794	3,623,739
7	2,575,941	12,531,567	652,409
8	1,270,508	4,016,181	787,107
9	2,486,241	21,177,837	1,127,568
10	2,191,943	12,985,426	1,046,819
11	1,897,645	4,793,014	966,070
12	1,090,925	7,616,823	281,520
13	3,358,741	7,217,293	2,225,783
14	8,076,258	3,764,003	6,888,492
15	1,340,542	4,272,573	401,681
16	977,064	4,365,095	429,469
17	613,586	4,457,617	457,256
18	1,080,013	8,622,903	395,513
19	1,546,439	12,788,189	333,770
20	1,069,980	4,588,936	4,499,213
21	1,314,542	13,560,333	637,979
22	2,114,559	26,474,033	2,296,707
23	4,634,036	18,756,326	2,353,462
24	1,914,422	10,515,160	442,306
25	1,162,875	6,904,352	724,797
26	3,087,726	8,523,070	1,223,388
27	4,704,555	3,546,972	703,018
Mean	2,388,886	9,800,770	1,844,091
SD	1671012.322	6194686.534	2673781.674
