# Supplementary material for: A novel algorithm for complete ranking of DMUs dealing with negative data using Data Envelopment Analysis and Principal Component Analysis: Pharmaceutical companies and another practical example
Source: PLoS One. 2023 Sep 1;18(9):e0290610. doi: 10.1371/journal.pone.0290610 (PMC10473491; doi:10.1371/journal.pone.0290610)
Supplement: S4 Table — (PDF) [file pone.0290610.s004.pdf]

S4 Table

Raw data of output indices for the pharmaceutical companies (2021)

Company ID	GPM	OPM	NPM	ROA	ROE	Reverse of P/E	Reverse of P/S	Reverse of P/B
1	30.990	21.510	49.760	24.910	50.220	0.129	0.260	0.292
2	59.780	44.360	29.120	21.770	52.850	0.081	0.278	0.188
3	45.160	41.310	23.350	16.250	45.380	0.047	0.201	0.112
4	49.535	45.510	33.060	30.185	65.615	0.104	0.289	0.157
5	53.910	49.710	42.770	44.120	85.850	0.162	0.377	0.202
6	39.080	34.340	20.470	10.490	19.450	0.136	0.667	0.746
7	24.780	16.590	32.930	17.540	53.780	0.125	0.382	0.233
8	47.800	45.140	30.560	28.150	56.740	0.121	0.395	0.233
9	53.060	48.850	38.190	47.900	108.100	0.185	0.485	0.220
10	38.660	31.295	18.140	23.440	53.935	0.099	0.610	0.426
11	24.260	13.740	-1.910	-1.020	-0.230	0.014	0.735	0.633
12	48.530	40.480	32.250	22.670	70.770	0.184	0.571	0.317
13	35.040	27.740	11.230	8.360	27.280	0.058	0.518	0.230
14	21.210	12.510	8.040	2.390	3.010	0.018	0.226	0.847
15	26.510	19.510	25.810	11.460	44.280	0.035	0.137	0.096
16	38.500	33.400	30.560	25.750	67.910	0.105	0.317	0.163
17	50.490	47.290	35.310	40.040	91.540	0.175	0.498	0.230
18	49.865	46.340	33.235	35.385	90.590	0.173	0.523	0.230
19	49.240	45.390	31.160	30.730	89.640	0.172	0.549	0.230
20	51.010	44.530	37.240	30.370	59.440	0.156	0.418	0.328
21	41.050	37.440	29.110	24.090	67.460	0.103	0.353	0.178
22	40.160	38.270	24.490	26.860	100.300	0.158	0.645	0.202
23	45.280	42.600	30.950	24.340	50.630	0.133	0.429	0.306
24	49.460	41.270	55.910	30.930	72.960	0.142	0.253	0.198
25	52.870	45.100	42.820	33.890	68.080	0.119	0.279	0.215
26	42.140	35.740	24.950	20.400	40.900	0.100	0.400	0.263
27	69.000	67.090	60.730	72.340	85.280	0.162	0.267	0.246
Mean	44	38	31	26	60	0	0	0
SD	11.09568	12.32158	13.20201	14.50819	26.72909	0.049157	0.153158	0.176431
